# 559. Keeping up with Local Bugs in OB: Antibiogram-Driven Antibiotic Guidance for Peripartum Infections

**DOI:** 10.1093/ofid/ofaf695.168

**Published:** 2026-01-11

**Authors:** Erika Orner, Christopher Vo, Jared Coe, Phyu Thwe, Wendy Szymczak, Rodney Wright, Mei H Chang, Inessa Gendlina

**Affiliations:** Montefiore Medical Center, Bronx, New York; Montefiore Medical Center, Bronx, New York; Montefiore Medical Center/Albert Einstein College of Medicine, New York, New York; Montefiore Medical Center, Bronx, New York; Montefiore Medical Center, Albert Einstein College of Medicine, Bronx, NY; Montefiore, Bronx, New York; Montefiore Einstein, Bronx, NY; Albert Einstein College of Medicine, Bronx, NY

## Abstract

**Background:**

Infection is the second leading cause of maternal death, commonly resulting from conditions such as chorioamnionitis, endometritis, and postabortal infections. The American College of Obstetrics and Gynecologists recommends a regimen of ampicillin with gentamicin, along with clindamycin or metronidazole, or vancomycin if beta lactam allergies are present. These combinations remain the standard-of-care antibiotic regimen but are largely informed by older data. More recent studies have demonstrated both efficacy and safety of alternative antibiotics including the usage of beta-lactams. In this study, we reviewed our local microbiology data and implemented a pathogen-directed, antibiogram-guided update to our institutional standard of practice. We developed an electronic health record (EHR) obstetric order set for our health system from this review.

**Figure:**
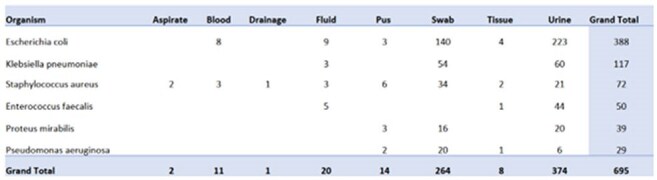
Total Cultures Evaluated Positive cultures of clinically relevant bacteria from Ob-Gyn patients who developed infections post-childbirth between 2020-2023.

**Figure:**
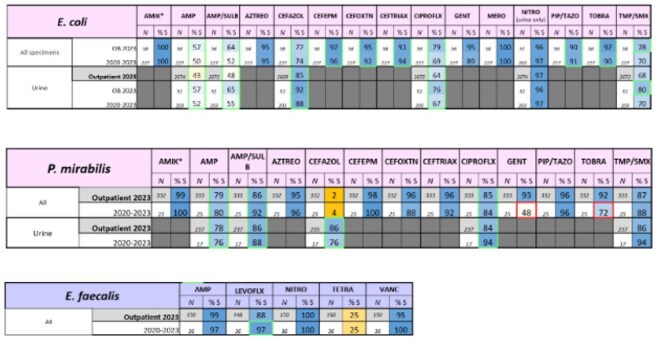
Updated Antibiogram Sample culture susceptibilities of clinically relevant pathogens were compiled across 3-hospitals within Montefiore Medical Center using samples from all anatomical sites

**Methods:**

We reviewed our local obstetrics and gynecology microbiology and resistance data for all clinical cultures obtained in our outpatient gynecology practices and inpatient obstetric triage and admission units. Based on culture results, we created an OB-specific antibiogram. We used it to guide and develop a new institutional antibiotic guidance and EHR obstetric order set.

**Figure:**
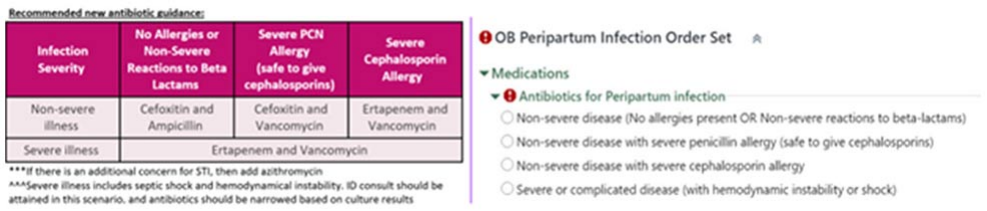
Peripartum Antibiotic Guidance

**Results:**

Between 2020-2023 we evaluated a total of 695 cultures from all anatomical sites. Total of 320 pathogens were speciated from non-urine samples vs 374 from urine samples. An antibiogram was produced based off these samples, directed towards clinically relevant pathogens. Based on the new antibiogram, antibiotic guidance was developed for patients with severe and non-severe infections. For non-severe patients, cefoxitin and ampicillin with the possible addition of azithromycin (for suspected concurrent atypical organisms) was recommended. For patients with severe infections, ertapenem and vancomycin with the possible addition of azithromycin was recommended.

**Conclusion:**

A new pathogen-directed, antibiogram-guided antibiotic treatment guidance was developed for treatment of peri- and postpartum infections. Clinician education and practice guidance is provided by leveraging EHR via peripartum order set.

**Disclosures:**

All Authors: No reported disclosures

